# Exploring Sustainable Approaches for Electronic Textile Products and Prototypes

**DOI:** 10.3390/s24175472

**Published:** 2024-08-23

**Authors:** Nishadi Perera, Arash M. Shahidi, Kalana Marasinghe, Jake Kaner, Carlos Oliveira, Rachael Wickenden, Tilak Dias, Theo Hughes-Riley

**Affiliations:** Nottingham School of Art and Design, Nottingham Trent University, Bonington Building, Dryden Street, Nottingham NG1 4GG, UK; nishadi.perera2020@my.ntu.ac.uk (N.P.); arash.shahidi@ntu.ac.uk (A.M.S.); kalana.marasinghe@ntu.ac.uk (K.M.); jake.kaner@ntu.ac.uk (J.K.); jose.oliveira@ntu.ac.uk (C.O.); rachael.wickenden@ntu.ac.uk (R.W.); tilak.dias@nut.ac.uk (T.D.)

**Keywords:** sustainability, electronic textiles (E-textiles), smart textiles, reusability, reparability, end-of-life

## Abstract

This research investigated the sustainability of textile garments with integrated electronics and their potential impact on the environment. The electronic textiles (E-textiles) sector is booming, with many advancements in E-textile product designs and construction methods having been made in recent years. Although there is a rapidly increasing interest in the reusability and sustainability of textiles, work towards E-textile sustainability requires further attention. Vastly different components are combined when constructing an electronic textile product, which makes it challenging at the end of the life of these products to dispose of them in a responsible way. In this study, a teardown analysis was conducted using a structured method, which first mapped out the interactions between each component of the product with the environment, followed by using Kuusk’s sustainable framework to analyze sustainable strategies. The research provides a unique contribution to transitioning sustainability theories into practical applications in the area of E-textiles, and the method proposed in this work can be employed in modifying electronics-embedded textiles to improve longevity and reduce the negative environmental impact. The work has highlighted key points of improvement that could be applied to a series of commercial E-textile garments, as well as a prototype E-textile device. Beyond this, the work provides a systematic approach for implementing new E-textile product designs that can evaluate overall product sustainability from the design stage to material selection, construction, and the planning of the commercial approaches of a product.

## 1. Introduction

This work presents a methodology for assessing the sustainability criteria for electronic textile (E-textile) products. Three commercially available garments were assessed using this framework in addition to a prototype E-textile garment.

Interest in complex electronic textile garments, including systems with embedded sensing and actuating capabilities, has grown in recent years [1]. The global E-textiles market is expected to grow from USD 1.8 billion in 2022 to USD 15.01 billion by 2030 [2]. Due to this increase in the market, there has been a growing focus on matters pertaining to E-textile sustainability.

Innovations in the field have seen capabilities including sensing, lighting, heating, and actuation incorporated into garments using a variety of methods such as surface-level electronics integration, attaching or bonding the electronics onto the textile, creating electronic functionality through the knitting or weaving of conductive yarns, printing electronics, and fiber and yarn level electronics component integration [3,4,5]. Different levels of integration will ultimately affect the ease of assembly, repairability, and recyclability. While products with attached electronics can often be separated easily, products where the electronics have some level of integration within the textile structure itself will introduce additional challenges. As good textile integration will affect user comfort, and therefore the desirability for the product, many have strived to achieve better electronics integration with textiles, and this trend is likely to continue. This work specifically focuses on the sustainability challenges related to electronics integrated within textile garments.

E-textiles are a hybrid product type at the intersection of textiles and electronics, and while there are several standards addressing the environmental and social impacts of textiles and electronics separately, specific standards for E-textiles currently do not exist. Textile waste is estimated at 92 million metric tons (Mt) annually, with 85% of this waste being dumped into landfills [6]. To reduce these large amounts of waste, standards have been developed to ensure that textile products are manufactured, used, and recycled or disposed of in ways that minimize harm to the environment and promote social responsibility. These include the GOTS (Global Organic Textile Standard, Global Standard GmbH, gemeinnützige GmbH, Rotebühlstr. 102, 70178 Stuttgart, Germany) [7] which covers the supply chain from harvesting organic materials to manufacturing, and the OEKO-TEX® 100 standard (OEKO-TEX^®^ Service GmbH, 2443, Fillmore St., Suite 380-1625, San Francisco, CA 94115, USA) [8] which addresses socially responsible manufacturing and eliminating harmful substances in textile materials. Other standards include the Bluesign^®^ System (Bluesign Technologies AG, Moevenstrasse 18, 9015 St. Gallen, Switzerland) [9] which focuses on sustainable textile production by managing the entire supply chain, and the Cradle to Cradle Certified™ system (Cradle to Cradle Products Innovation Institute Inc., 475 14th St Ste 290, Oakland, CA 94612, USA) [10], which evaluates products for their sustainability across different categories. 

The Sustainable Apparel Coalition (SAC, 82, 2nd St, San Francisco, CA 94105, USA) is a global alliance of textile, apparel, and footwear companies working to reduce the environmental and social impacts of these products [11]. They have developed a tool, the Higg Index [12], to measure the sustainability performance of products. The Higg Index comprises five tools that can measure the social and environmental impact on the value chain and economic viability. It provides a comprehensive assessment of a product’s lifecycle impact. The impact is measured in five major areas, covering global warming, water scarcity, nutrient pollution, abiotic resource depletion, and chemistry. A score is assigned to each material according to the impact [13]. By using the Higg Index, companies can make informed decisions to enhance sustainability in their products and operations. 

These frameworks help support the textiles and apparel industry to operate in an environmentally friendly manner. It is helpful for the industrial partners and consumers to adhere to these standards as they are well-defined and practiced across the platform. Hence, these tools foster a more sustainable textile industry. 

The electronics industry also produces significant waste, with E-waste expected to reach 82.34 million metric tons (Mt) by the end of 2030, an increase of 37.64 million Mt since 2016 [14]. The Waste Electrical and Electronic Equipment Directive (WEEE) has been developed to limit E-waste and it is widely used to set collection, recycling, and recovery targets for electronic waste in many countries, including the countries of the European Union and the United Kingdom, where adherence is stipulated by law. This directive outlines that manufacturers are required to consider reparability and recyclability when designing products and to take responsibility for the collection and recycling of the products at the end of their useful lives. This creates the need to implement take-back schemes from the consumers and the associated infrastructure. WEEE-compliant products are labeled with the crossed-out wheeled bin symbol, indicating that the product should not be disposed of with general household waste and must be processed in facilities designated to handle electronic waste.

E-textiles can sometimes be classified under one of the WEEE categories due to the presence of electronic parts and specifies the conditions under which products fall under its guidelines, making a distinction based on whether the electronic functionality is fundamental to the product’s purpose or merely supplementary. It should be noted that many E-textile products on sale do adhere to the WEEE regulations, for example, “Homefront Electric Blanket” aligns with the WEEE guidelines, and the brand has an in-store electronics recycling protocol [15]. Manufacturers can also design E-textiles with removable electronics to avoid compliance with WEEE directives for the textile portion of the product. Once the electronics are removed, the product may not be treated as E-waste and subsequently a significant number of E-textiles are disposed of as regular textile waste, not as electronic waste [16,17,18]. Beyond this, consumers might simply not understand what to do with faulty or unwanted E-textile products and dispose of them in regular waste streams. Consequently, these products often end up in landfills rather than being properly recycled through WEEE waste streams.

Developing sustainable E-textiles requires a significant initial investment in research and development and such system integrations may not be scalable yet. Some of the development approaches can lead to design limitations which hinder the manufacturer from achieving the desired product. Brands have to invest in implementing take-back schemes and making consumers aware of such new practices [19]. However, manufacturers and brands should foresee the long-term benefits of implementing sustainable practices in their businesses as it provides a competitive advantage with growing consumer concerns about the environmental impact of products. The eco-conscious customer base is growing and taking early actions will future-proof the businesses. 

Importantly, the effective recycling of complex E-textiles requires the development of specialized recycling infrastructure capable of handling both the textile and electronic waste [20].

The E-textiles product category supports some of the global sustainable initiatives, such as COP28 [21,22]. COP28 sets out targets and strategies to address global climate change by enhancing energy efficiency, reducing waste, and enabling sustainable product development and consumption. For example, some of the ongoing research on electronic textile energy harvesting through the incorporation of solar cells into apparel may lead to self-generating energy capabilities [23]. 

The evaluation of the sustainability of E-textiles has received relatively little attention in the literature, with some notable exceptions. Kohler et al. have explored waste-preventative eco-design methods for E-textiles [24]. The study focused on the importance of identifying methods of E-textile construction to facilitate recycling, compatible standards in this space, and design for disassembly (DfD) strategies: it is important to implement these design schemes when beginning to design any product to save time and money. Other designs for disassembly research involving E-textiles have been conducted by researchers such as Saunders, Casciani, and Chen, who have demonstrated the practicality of DfD through their work [25,26,27]. Saunders has investigated the potential for establishing policies in waste management and legislative solutions to manage future E-textiles waste in an effective way [25]. Jabbour et al. [28] have discussed circular economy models for E-textile businesses in their work and illustrated a framework highlighting a data-driven approach for managing the lifecycle of E-textiles. Further, bodies such as IPC International Inc. (Bannockburn, IL, USA) are implementing standards on design, quality, and reliability requirements for E-textiles to be in line with possible future sustainable standards [29].

To understand the recyclability and sustainability of E-textile products, good current practices, and areas of improvement for current products, the disassembly and analysis of E-textiles can be a beneficial method. Hardy et al. have previously conducted a teardown workshop to assess three existing E-textile garment products, where they qualitatively analyzed the construction of the garments and offered suggestions for improving their repairability [30]. The work highlighted the importance of proper labeling on the product and the need for regulations in the industry.

Kuusk has also examined E-textile sustainability and has compared E-textiles with the conventional fashion cycle [31]. Their study proposed a sustainability framework that can be used in future works to analyze the impact of this product category on environmental, economic, and social pillars. In order to arrive at this framework, Kuusk interviewed experts in the sustainability field, captured their responses, and then analyzed and categorized them to create a meaningful framework.

The above-mentioned works highlight the difficulty of repairing electronics-embedded textiles. Some fiber-level electronic component integration makes it complicated to separate electronics from textiles and hence reparability becomes very complex [32]. This leads to the need to implement sustainable considerations from the product design stage itself. 

This work builds on the existing literature to further understand good practices and possible points of improvement for existing electronic textile products. Initially, a teardown workshop was carried out, similar to the method employed by Hardy et al. [17]. A design structure matrix was then used to understand the interdependencies of each component and assess their impact on the environment. Finally, the Kuusk framework was applied to each product to analyze possible areas of improvement to enhance the sustainability of the products.

The analysis of the commercial products was followed by a similar teardown of an E-textile prototype developed by the research group. The same analysis was applied to try and better establish ways of improving the prototype’s design and construction to enhance its sustainability credentials.

This study provides a new integrative approach to assessing the sustainability of electronics-embedded textiles, which is a complex product category. The information gathered through the teardown workshop was systematically analyzed, which is a unique procedure that can be employed in other future studies as it provides a granular level understanding of how each component of a product impacts the environment. Although there have been some teardown workshops carried out on E-textiles, systematic further analysis of the components of the products and their interactions has not been conducted in earlier works. Further, this work uses the developed approach to improve a prototype E-textile design. This transition from theory to practice is a unique approach, offering knowledge on how sustainability improvements can be implemented in real-world products. This research bridges the gap between sustainability theory and practical applications in the E-textiles area. The knowledge can further enhance sustainability in this growing industry.

## 2. Materials and Methods

### 2.1. Product Selection

**Figure 1:**
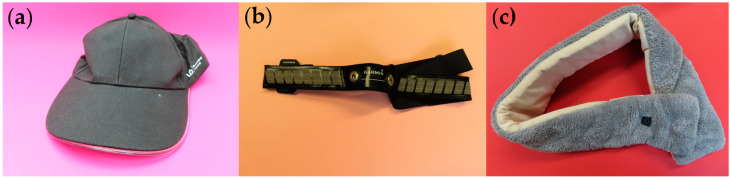
Commercial E-textile garments that were used for the tear-down analysis. (**a**) Heated scarf. (**b**) Light-up cap. (**c**) Heart rate monitoring band.

The three commercially available electronic textile garments were selected for the teardown workshop following a review of products on the market. It was desirable to select products that represented the key commercial product areas (lighting, heating, and sensing) and a mixture of electronics integration techniques. Further, only E-textile garments were considered as the sustainability considerations for other product types may have been different. With these criteria considered, the following garments were selected:

- Heated scarf by GLAITC (full company details unavailable, purchased from Amazon). This product represented an electronics integration technique where conductive elements were held between two textile layers (Figure 1a).
- A light-up cap by Illuminated Apparel (London, UK). The product was purchased from Amazon. This represented an E-textile where the core electronic functionality (the light-up band) had been attached to the surface of the textile, with wiring running inside of the textile (Figure 1b).
- A Garmin HRM dual-band (Garmin, Schaffhausen, Switzerland), which is a heart rate monitoring strap. This product was purchased from Amazon. This gave an example of an E-textile where a key component, here the electrodes, was created through the weaving of conductive elements (Figure 1c).

The authors concede that this is not a comprehensive list of commercially available garments, however, this small cross-section was deemed appropriate for testing the proposed methodology. Knowledge of the historic developments and market for the field [1], combined with a brief analysis of currently available products indicated that heating, lighting, and heart rate sensing remain key product areas in this sector.

### 2.2. Teardown Workshops

A two-hour teardown workshop was organized at the Nottingham School of Art and Design, Nottingham Trent University (UK). There were twelve participants in the workshop, including two facilitators, who were recruited from University staff and postgraduate students. The participants included experts from a range of relevant areas including electronic textiles, fashion, sustainability, and medical devices. This cross-disciplinary participation helped to ensure that opinions were impartial and that valuable viewpoints from people with different expertise could be collected. To hold the workshop, ethical approval was obtained from the Nottingham Trent University Schools of Art and Design, Architecture, Design, and Humanities Research Ethics Committee. Informed consent was obtained from all of the participants before the study.

Physical notes were taken during the workshop and the discussion from the workshop was recorded and transcribed. These notes and the discussion were used to inform the teardown analysis below (all meaningful information is included in this article).

The workshop started by acknowledging common challenges in the E-textiles space when considering sustainability, the requirement for standards outlining post-use, and sustainable manufacturing processes for E-textiles. The aims, objectives, and workshop structure were also explained. Participants were asked to think about what was/could be taken or adopted by E-textile manufacturers when considering the end of the lifetime of a product, the efficiency by which the electronic components of the E-textiles could be separated and potentially recycled, and strategies that could be implemented to improve reusability, and the recyclability potential of the electronics embedded in the textiles.

All three products were presented to the participants and the teardown was conducted for each product. Further, participants were asked to look at and discuss sustainability considerations related to a prototype E-textile (a temperature sensing sock), and feedback was collected. The teardown of the sock was conducted as part of a separate teardown activity with two of the authors carrying out the teardown.

### 2.3. Analysis of Electronic Textiles

E-textiles were analyzed using three techniques. Firstly, the teardown workshop was carried out, and participant feedback was collected (as discussed above). With knowledge of the constituent components of each E-textile, an interaction matrix was prepared (based on a design matrix), where the interaction between the components of the products and their potential impact on the environment was explored. Finally, Kuusk’s framework was applied following the method outlined in the literature [31], where the products were analyzed within the context of Kuusk’s eight sustainability qualities (SQ), describing eight principles relating to environmental, social, and economic sustainability:

- SQ1 Minimizing Consumption;
- SQ2 Controlling Energy and Chemical Use;
- SQ3 Developing Constantly;
- SQ4 Caring for Longevity;
- SQ5 Supporting Meaning Creation;
- SQ6 Updating the Product;
- SQ7 Empowering Positive Emotions;
- SQ8 Building Relationships.

The Sustainability Qualities framework was employed to organize and expressways that modifications to the product and construction process could help the product better align with sustainable strategies. 

### 2.4. Electronic Textile Prototype

**Figure 2:**
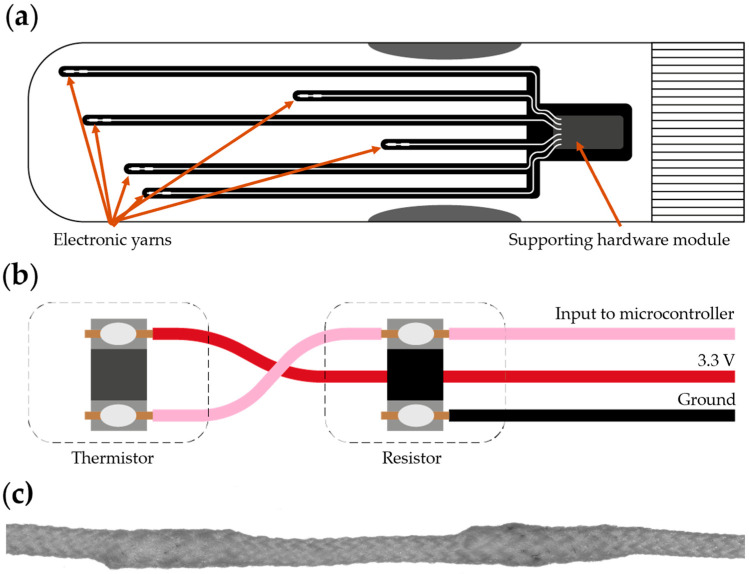
Temperature sensing sock prototype. (**a**) Schematic of the base of the sock showing the locations of the temperature sensing E-yarns and supporting hardware module. (**b**) Schematic showing the wiring of the embedded thermistor and resistor inside of the E-yarns. (**c**) Microscope image of a temperature sensing E-yarn. This image has been stitched together from two images.

The prototype E-textile analyzed in this work was a temperature sensing sock that was created using electronic yarn (E-yarn) technology (Figure 2). Earlier iterations of this prototype have been shown elsewhere in the literature [33]. This version of the sock, produced in late 2021, incorporated six temperature sensing electronic yarns with embedded temperature sensors (10 kΩ NTC thermistor part number: NTCG103JX105DT1; TDK Corporation, Chuo City, Tokyo, Japan) and resistors (10 kΩ resistor part number: CRCWO40210KOFKED, Vishay Intertechnology, Malvern, PA, USA) to complete the voltage divider (Figure 2b). These were crafted by first taking commercially available Litz wires (outer diameter = 254 µm; BXL2001, OSCO Ltd., Milton Keynes, UK) and soldering these onto a thermistor and a resistor using a contact soldering iron (Antex XS25; Antex, Plymouth, UK) and lead-free solder (o.d. = 0.010 mm). The discrete components were then encapsulated within a small resin pod (approximately 4 mm in length, 1 mm in diameter; Dymax Multi-Cure^®^ 9001-E-V3.5, Dymax, Torrington, CT, USA). Each of these soldered filaments where then covered with 24 ends of 167 dtex/48 filament polyester (J. H. Ashworth & Son Ltd., Hyde, UK) using a braiding machine (Herzog RU 1/24-80; Oldenburg, Germany). A sock was knitted using a grey 167 dtex/48 filament polyester yarn on a Stoll ADF 3 E7.2 flatbed knitting machine (Lengede, Germany). Channels were incorporated on the base of the sock in the knit using a tubular warp knit technique to allow for the careful positioning of the temperature sensing yarns and to facilitate the yarns removal at end-of-life, or repair if a breakage occurred. A single stitch was used to secure the E-yarns once it was inserted into the sock. The ends of the E-yarns were then soldered onto a receptacle connector (part number: 20021311-00010T4LF; Amphenol ICC, Wallingford, CT, USA) and the connections and connector were consolidated using a heat shrink: This would be used to connect to a removable supporting hardware module that incorporated a power supply, Bluetooth connectivity, and the sock’s controlling electronics. The module would sit within a pocket inside of the knitted sock when in use. The supporting hardware was constructed from an Adafruit Itsy Bitsy nRF52840 Express Microcontroller (New York, NY, USA), power management board (Adafruit LiIon/LiPoly Backpack), a sliding switch (model SS 12; NKK Switches, Scottsdale, AZ, USA), Rechargeable Lithium Polymer Battery (3.7 V, 40 mAh; UFX 401215, Guangdong, China), connector, and wiring comprising multistrand copper wires and Litz wire. The module was encased within a silicone with Shore A10 hardness (Platsil Gel 10, Polytek Development Corp., Easton, PA, USA) to protect the electronics and to minimize the effects of the electronics on the comfort of the wearer.

## 3. Results and Discussion

### 3.1. Heated Scarf

**Figure 3:**
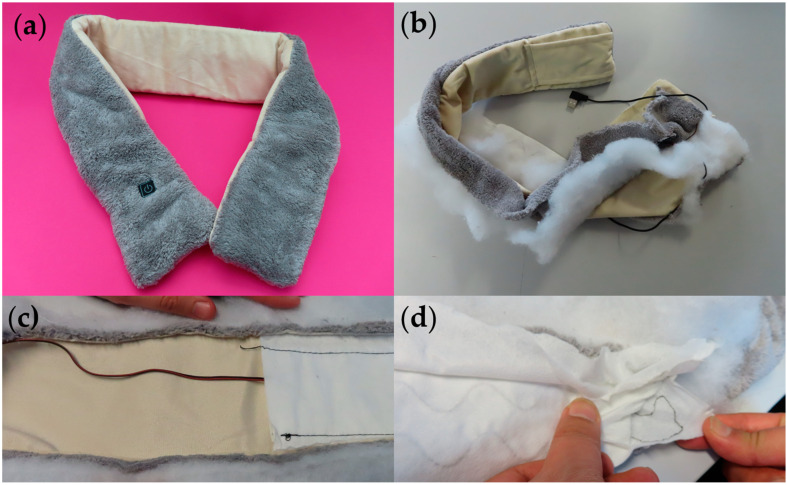
Heated scarf. (**a**) Product prior to the teardown. (**b**) Product following disassembly. (**c**) Detailed photograph of the wiring connection. (**d**) Detailed image of the conductive heating element.

The heated scarf was an active heating product where integrated electronic components were used to achieve the heating functionality (see Figure 3a). The product was purchased on 21 September 2023 for GBP 4.59. The product was delivered in a plastic bag without any branding information. There were no additional leaflets or an instruction manual in the package. There was a USB A-type connector attached to the scarf to power the conductive wires in the product. The scarf could be activated via a mechanical switch, enclosed in a silicone mold. There was some information about the product on its Amazon webpage, where the product was purchased, which covered the type of materials used, the construction of the mini pocket that holds the power supply unit, and some promotional details to attract customers. There were three heating levels corresponding to 30 °C, 40 °C, and 50 °C, which could be adjusted using the small power button on the scarf: this was noticed during the initial handling and inspection of the product. The webpage claimed that the product was washable after the removal of the power source, however, there were no indications on the number of wash cycles it can withstand. Washing test standards were not mentioned on the product webpage and proof of the wash durability was not provided. The longevity of the product may be affected due to washing, but a full analysis of this was not possible given the limited information provided. The website did not mention any regulatory compliances in terms of the product’s functionality or sustainability. Consumers were not advised on product disposal methods and how this product should be treated at the end of its lifecycle. 

During the teardown, it was observed that the outer layer of the scarf was made out of synthetic brushed fabric and that the product was stitched along the perimeter with closed stitches. The USB cable, which was connected to the scarf, was required to plug the device into a power supply. There was a hidden pocket constructed on one side of the scarf to place the power supply, however unfortunately the power supply itself was not included with the product. From the information available on the webpage, it was evident that any suitable power bank or a portable charger of around 5000 mAh could be used to power the scarf. There were no care labels on the article so a consumer would not be able to easily find information regarding washability and ways of charging the device, and would instead have to refer to the product’s webpage, which was identified as a major drawback during the workshop.

The product was torn down step-by-step, first by cutting along the hem to reveal the materials inside (Figure 1b). The electronic components were well hidden inside the outer brushed layer and flexible conductive threads and heating conductive elements were attached to a nonwoven fabric layer through basic embroidery. The conductive wires consisted of metal (believed to be copper) threads to allow for heating when a current is passed through the wires. 

During the teardown, it was observed that different types of nonwoven materials were used in the internal structure, a stiff nonwoven layer held the conductive wires, and the wires were then covered with another less stiff nonwoven fabric. These two layers were glued together, probably to ensure the water resistance claimed on the website. Although this structure was assumed to protect the electronics, the level by which the layers were secured may not have been robust enough to make it water-insulated. There is, therefore, a possibility of damage occurring to the circuitry during a wash cycle, or faults occurring in wet conditions. Any breakages would make the product obsolete as there are no means of repairing it, and the consumer would treat the product as general waste as there are no alternative instructions given.

The interaction matrix for the scarf showing the interaction between the components and their potential impact on the environment is shown below in Table 1.

**Figure 4:**
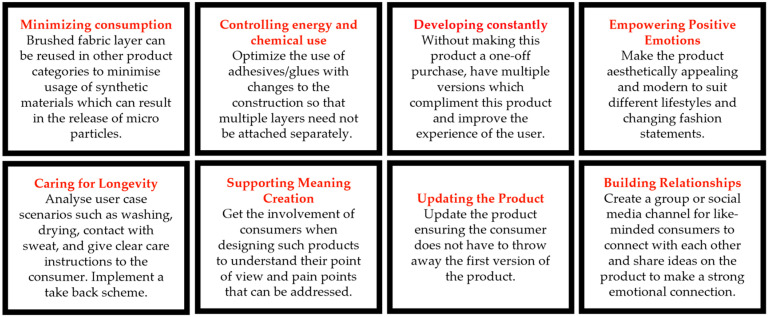
Kuusk’s eight sustainability qualities applied to the heated scarf.

The main drawback of this product was the lack of information on the care label for the safe disposal of potentially harmful items. There was no easy means to segregate the product into textile and electronics waste. The low price point of these types of products motivates consumers to purchase more of these items and dispose of them as soon as they stop functioning. The circuitry arrangement and the lack of attention to detail when insulating the electronics from water ingress led to a strong assumption that the product may have a very short lifecycle. The construction of this product consisted of glue, stitching, and embroidery, which made it challenging to easily dismantle the electronic component from the textile or the nonwoven component. So, even if a take-back scheme was implemented, too much manual labor would be required to segregate the components, making such a scheme very costly to a company. Figure 4 shows the product analyzed using Kuusk’s eight sustainability qualities including suggestions on how to improve the product.

### 3.2. Light-Up Cap

**Figure 5:**
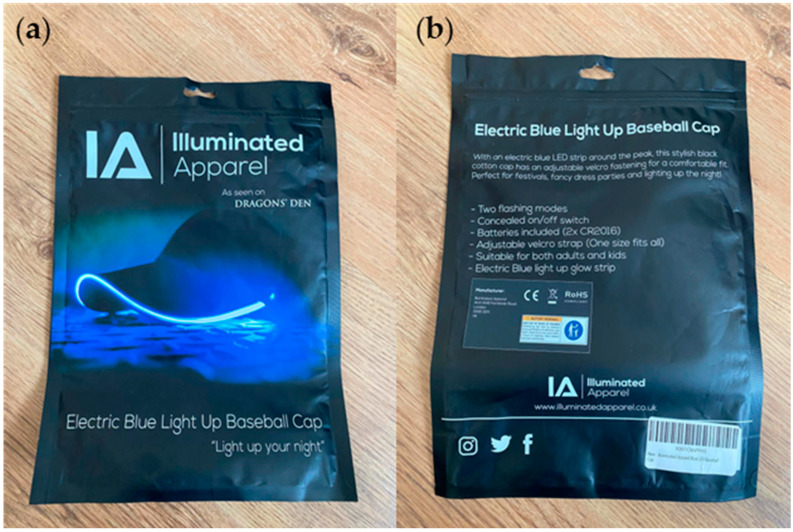
Photograph of the packaging of the light-up cap. (**a**) Front of packaging. (**b**) Rear of packaging. Note the inclusion of the WEEE symbol on the back of the packing.

The light-up cap can be categorized as a fashion accessory at an affordable price point, costing GBP 12.95 when purchased on 21 September 2023. The product was delivered in company-branded packaging, which had information on the brand and the use of the product, as shown in Figure 5. The package consisted of the light-up cap and 2 × CR2016 batteries (3 V lithium batteries). Extra batteries can be purchased from any vendor once the provided batteries run out. The product did not have any care labels or leaflets outlining instructions to the consumer. The website describes how the product works. There were three light settings on the cap: a fast flash, a slow flash, and a solid color option. There was a small switch concealed in the brim of the cap. 

**Figure 6:**
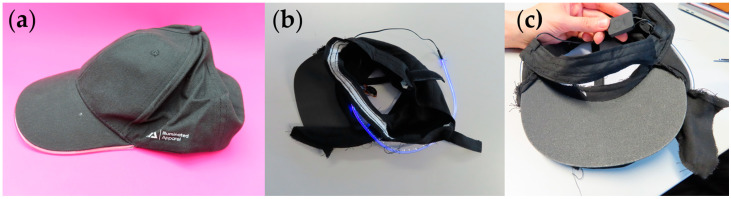
Light-up cap. (**a**) Product prior to teardown. (**b**) Product following disassembly. (**c**) Detailed photograph of the supporting electronics and power supply.

The materials and construction methods were identified during the teardown (Figure 6). The textile components of the cap were made of 100% cotton, so the outer materials could be recycled. The light-up strip was made of LED lights integrated into a film that was placed along the perimeter of the cap brim. The cap brim consisted of a stiff nonwoven layer to provide shape and stability to the brim structure. The LED strip was mounted onto this material through stitching. There were wires connecting the LED strip and the battery pack. All of the wires and electronic components were well hidden between different layers of materials. After connecting the battery to the exposed end of the wires, the battery could be hidden inside of a small opening on the cap brim. The website did not make any claims about how water may affect the functionality of this cap. Once the internal construction was studied, it was recognized that the circuit was not well insulated so there was a possibility for water to damage the circuitry and stop the cap from functioning. This scenario would directly affect the product’s lifecycle, and the consumer would dispose of the product once it had broken.

The interaction matrix for the cap showing the interaction between the components and their potential impact on the environment is shown below in Table 2.

**Figure 7:**
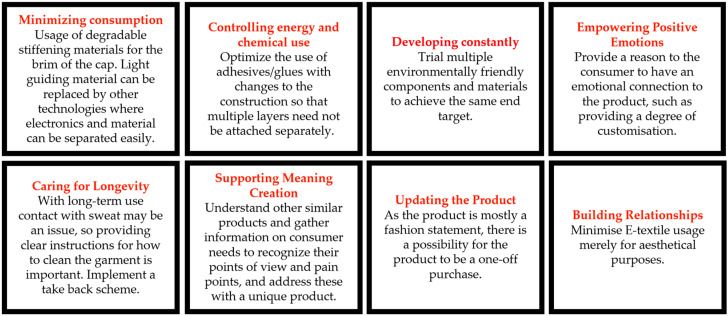
Kuusk’s eight sustainability qualities applied to the LED light-up cap.

The light-up cap had similar challenges and drawbacks to the heated scarf product, even though this company did provide further information on disposal (WEEE labeling). Adhesive material between several layers of the product makes it very challenging to separate each layer and even if some of the layers are biodegradable, it would not be a possibility due to them being attached together. The lack of sophistication in the electronics arrangement and encapsulation may impact the product’s lifetime negatively. The fact that it is considered as an aesthetic item would limit a consumer from trying to fix the product and reuse it if it became damaged; the relatively low price point may also discourage repair. The emotional bond to the product is important for a consumer to actively use a product for longer periods of time without making it obsolete and replacing it with another item, and the clear company identity may help the product in this respect. The inclusion of a care label would provide more awareness to the consumer regarding how to maximize the product’s lifetime. Kuusk’s eight sustainability qualities applied to the light-up cap are shown in Figure 7.

### 3.3. Heart Rate Monitoring Strap

The Garmin Heart Rate Monitoring dual band was capable of taking a user’s heart rate (using an electrocardiogram), with the data displayed via an app by wirelessly connecting the band to a smartphone. The product cost £47.82 (purchased on the 21 September 2023), making it the most costly product examined. Garmin is also a well-established brand and as such would be under pressure to be socially and environmentally responsible by its competitors, consumers, and regulatory bodies.

The product was packaged in a branded box containing the soft strap with electrodes, a strap extender, and an instruction manual. The soft strap has a detachable supporting hardware module (shown below). Garmin’s official website was a key source for information about the product and company sustainability policies. The company claims that the strap is washable and the battery that can last up to three years, and the website and the manual clearly illustrate wash instructions. The strap should be hand-washed to avoid the build-up of sweat and salt, which can permanently damage the heart rate monitoring electrodes. A tiny amount of detergent has to be used when hand washing and the strap should be dried by hanging it up or laying it flat. This limitation might hinder the lifetime of the product as having to hand wash a garment may be viewed as a burden by a consumer. The website provides information on the technical specifications of the device, compatible devices, and instructions on how to use the device. The manual mentions that coin cell batteries are used in the product and how they should be safely disposed of. 

**Figure 8:**
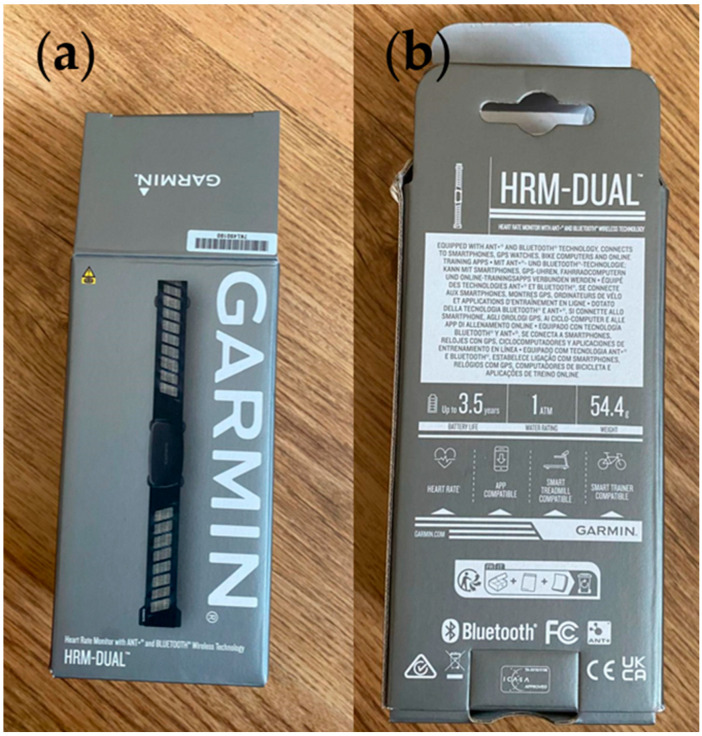
Packaging for the heart rate monitoring chest strap. (**a**) Front of packaging. (**b**) Rear of packaging. The WEEE symbol is clearly shown.

The company also outlines its sustainability initiatives on its website and how they have considered and implemented steps to improve sustainability across its product range. The company has received ISO 14001 [34] “Environmental Management” certification, which deals with processes within the organization related to the design, manufacture, and distribution of its products. The company also discusses sustainable approaches they consider in their processes. They ensure that their packaging is recyclable and made in a way that has minimal impact on the environment. Their products and packaging comply with global regulatory requirements for substance restrictions and the website claims their packaging is made of more than 80% recycled material. The packaging received with this product is shown in Figure 8.

Garmin is compliant with the EU directive on waste electrical and electronic equipment (WEEE, Rue de la Science 23B—1040 Brussels, Belgium): they strive to reuse electronic components and have partnered with electronic waste recyclers for items that cannot be reused. They also offer the customers the opportunity to recycle their old electrical items when purchasing a new item from them. This take-back scheme provides the consumer with the chance to participate in making a more sustainable world. Although there were not any specific care instructions mentioned on the product label, the supporting hardware module and the instruction manual included in the box specifically showed a crossed-out wheelie bin symbol which indicated the adherence to WEEE standards. This showed that the module has to be treated as electronic waste and not to be thrown away with general waste.

**Figure 9:**
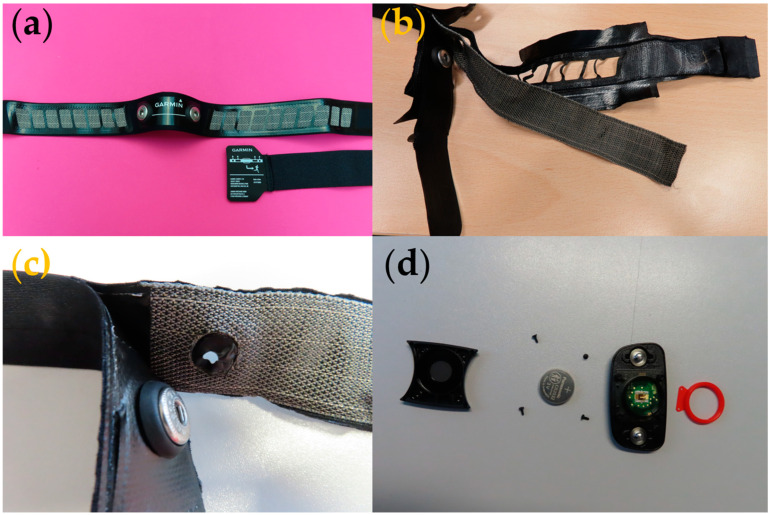
Heart rate monitoring strap. (**a**) Product prior to teardown. (**b**) Product following disassembly. (**c**) Detailed photograph of snap fastening connectors. (**d**) Photograph showing the disassembled supporting hardware module.

During the teardown of this product (Figure 9), the group appreciated that the product came in a paper box which can be easily recycled. While there was polyethylene packaging inside of the box with the strap packaged inside of it, it still adhered to the company’s claim of using recyclable material for 80% of the packaging material. 

How the device worked was understood and discussed before the teardown of the product. Each layer was taken apart until the circuitry inside was revealed. Each element was noted down in the interaction map for further analysis.

It was observed that there were two connectors to attach the Garmin supporting hardware module onto the strap (Figure 9c). The module allowed the strap to connect to a mobile phone app via Bluetooth. The company uses a system called ANT+, which is widely used in fitness technology, so there is a low probability of the platform becoming obsolete in the near future. However, the device pairing protocol and compatibility should be maintained and supported in new designs as well.

Following the teardown, the interaction matrix for the heart rate monitoring band showing the interaction between the components and their potential impact on the environment was developed, as shown below in Table 3.

The heart rate monitoring band was a well-designed product compared to the other two products investigated and the electronics were well-secured and insulated, which would enhance the product’s longevity. The instructions provided by the company on disposal and recycling schemes are very helpful to the consumer and it should motivate the consumer to participate in these schemes. However, the product’s construction and the use of some materials may make it difficult to recycle, as shown by the interaction matrix above.

The outer fabric layer of the strap is made of polyester, and this can be recycled if the fabric can be easily disassembled from the other components. The woven fabric strip holding the electronic component (the woven electrodes) can also be recycled or reused for another purpose if the electrodes can be removed. The snap buttons used in the product are metal buttons that can be disposed of as metal waste or reused for other garments or accessories. The supporting hardware module can be removed from the garment and can be easily treated as electronic waste. The coin cell battery embedded in this module can also be removed and the company has given guidelines on safely disposing of the battery by treating it as electronic waste under WEEE.

There are, however, multiple elements of the product that negatively affect the environment. The elastic strap is a common challenge in the textile industry because it is difficult to recycle textiles with elastomeric yarns or compounds in them. Heterogenous materials, especially where they have included an elastomeric, are generally difficult to reuse and recycle. There are plastic strap adjusters on the chest strap to make it fit the body: These plastic pieces are not degradable, and they would have to be disposed of in landfill. Replacing these plastic adjusters with another material or a different construction would be beneficial in terms of sustainability. One of the major complications with this product is the addition of vinyl polymer and waterproof membrane layers to prevent water from reaching the electronics. However, it is necessary to have these layers to ensure that the product functions correctly for a longer time period. Another major challenge is the use of multiple adhesive layers inside the product. The interaction map illustrates that the adhesive is in contact with almost all of the inner layers of the band, bonding them all together. This adhesive bond makes it very difficult to separate the materials when trying to recycle or reuse the device. Elements such as the woven fabric strip, which normally could be easily recycled, cannot be recycled in this case due to the adhesive glue on the material.

**Figure 10:**
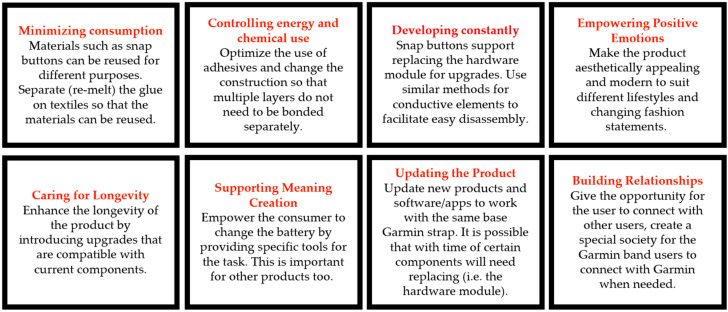
Kuusk’s eight sustainability qualities applied to the heart rate monitoring band.

The Sustainability Qualities framework was subsequently employed to organize and expressways for the product to better align with sustainable strategies (Figure 10).

### 3.4. Prototype Temperature Sensing Sock

**Figure 11:**
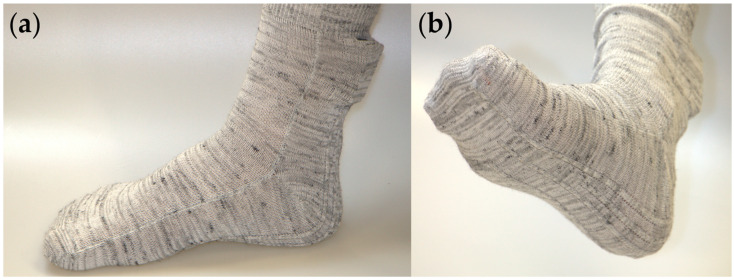
Temperature sensing sock prototype created using electronic yarn technology. (**a**) Side view. (**b**) Bottom view showing the knitted channels into which the E-yarns are inserted.

The prototype temperature sensing sock was constructed using E-yarn technology (Figure 11). As this prototype was built by the research group (the Advanced Textiles Research Group at Nottingham Trent University), the group had detailed knowledge about the materials used in each step of the sock’s construction. The objective was to identify the improvements that could be made to this E-yarn-based prototype and enhance future E-yarn prototype designs, to make them more sustainable (with the ultimate goal of ensuring that the final products created with the technology had good sustainability credentials).

**Figure 12:**
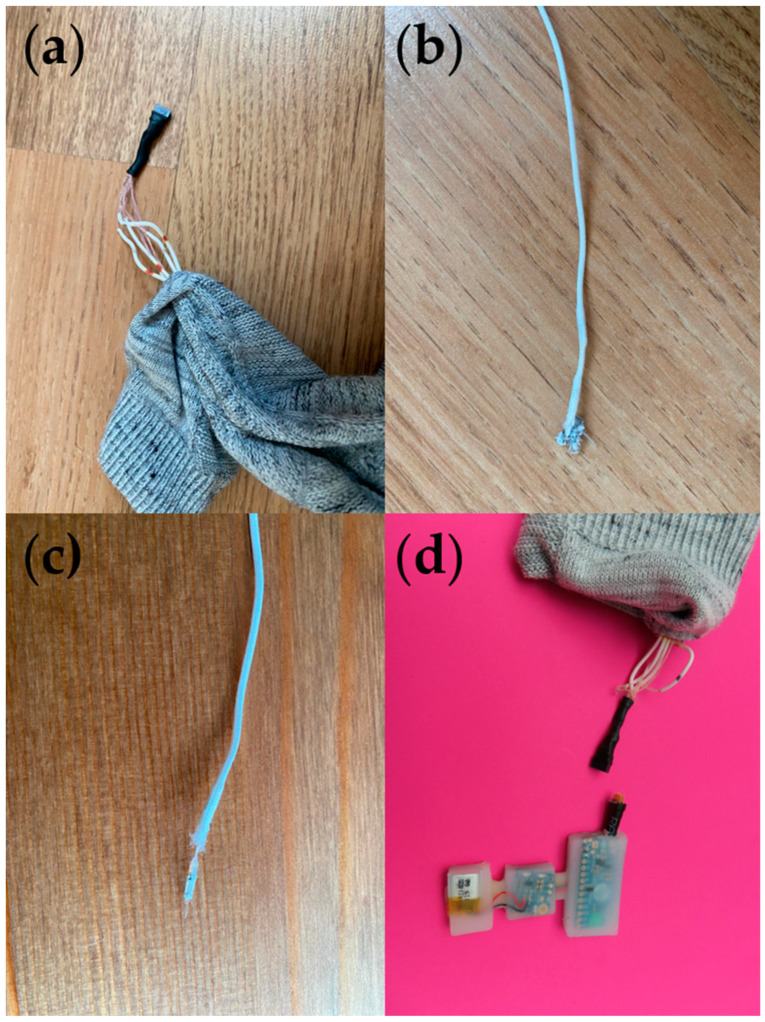
Temperature sensing sock prototype. (**a**) Image showing the connector to the supporting hardware module. (**b**,**c**) Images of the embedded electronic components. (**d**) Image of the supporting hardware module.

Feedback regarding the temperature sensing sock from the teardown workshop was limited, with comments largely focusing on the repairability of the supporting hardware module. The sock was subsequently torn down in a similar way to the other products (Figure 12).

**Figure 13:**
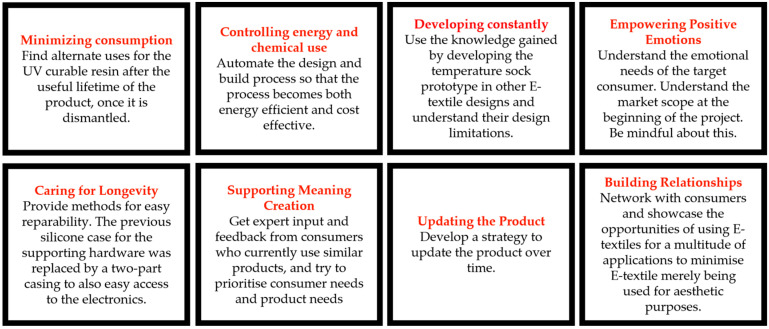
Kuusk’s eight sustainability qualities applied to the temperature sensing sock prototype.

As with the products, an interaction matrix for this prototype was created to understand the component interaction (Table 4). Once the analysis was completed, Kuusk’s model was employed to further analyze modifications to be made to the prototype design (Figure 13). 

The supporting hardware model was seen to be the main factor to redesign to improve the longevity of the product, as identified during the initial teardown workshop, once it is commercialized. The existing module had a soft silicone casing, so it would be difficult to repair the module if it became damaged or if the embedded battery failed. Other considerations that would need to be taken if this prototype were to be developed into a product included the need for after-sales services for minor repairs, and take-back schemes to separate electronics from textiles materials for recycling and reuse. It should be noted that during the teardown the removal of the E-yarns was relatively easy, showing the effectiveness of using knitted channels for ease of removal.

**Figure 14:**
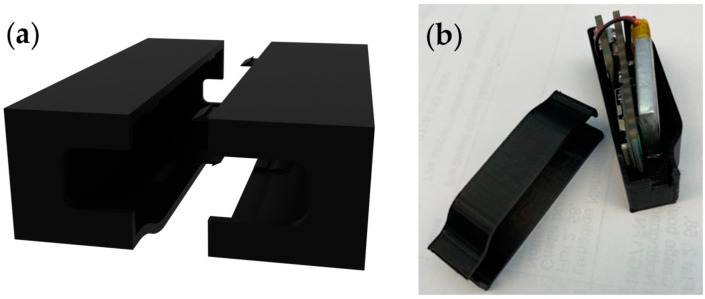
New temperature sensing sock prototype hardware module. (**a**) Three-dimensional render of the casing. (**b**) Complete module.

Based on these findings, the supporting hardware module was redesigned to provide easier access to the internal electronics, facilitating easier repair. This was achieved by introducing a two-part casing to hold the supporting hardware. This would also allow for easy disassembly at end-of-life. As seen in Figure 14, the two parts of the module, which were 3D printed using ABS, could easily clip together. The group has subsequently upgraded their hardware module designs further, however, the principle of having a module that can be opened and designed for easy disassembly has remained.

### 3.5. Guidelines for Designing New E-Textile Products

This study followed a structured method of analyzing and evaluating the sustainability of E-textile products. The method was applied to three different commercially available products and an E-textile prototype, providing case studies for using the framework. Although this method was applied to already designed and developed products or prototypes, it was understood that the proposed method can, and should, be iteratively used in the product design stage to understand and assess end product sustainability in an effective manner. 

Firstly, the interaction map identifies how each component of a product interacts with one another and the environment, and how essential they are for the product functionality: This lays the foundation for understanding the product’s lifecycle. This insight is important for designers as it provides information on which components and materials to select in order to have a minimal negative impact on the environment. When developing prototypes in the design stage, this may highlight the need to trial alternative materials for the essential components and identify areas where the product is not very sustainable. For example, multiple adhesive layers were identified as an issue for all three of the commercial products which limited the recyclability of the products. The map provides a holistic view of a product which can guide designers and innovators to consider different solution hypotheses in an environmentally conscious manner. 

Once these component limitations were identified, applying Kuusk’s sustainable framework provided great support in evaluating different alternate solutions, which led to the overall sustainability of the E-textile product. The modifications suggested to the product can be systematically reviewed to realize how this would have a positive impact on sustainability. The principles, including correct material selection, constant development, and care for longevity, would massively help in designing a product that reflects sustainability criteria. 

It is suggested that this framework should be employed during the early stages of product development. It would be ideal to use it during the E-textile product design stage, which is a suitable point to address the overall sustainability of the designed product where proactive decisions can be made to change materials, components, structures, and the construction of the product. The method can be used iteratively during the design stage, re-examining the maps as components change. The time spent on this approach during these initial stages of a product’s development will reflect well in the later stages of the design process and will help ensure that the resultant product has fully considered sustainability in its development.

## 4. Conclusions

This study evaluated challenges faced by E-textile products in terms of long-term sustainability with a focus on three commercial E-textile garments, and one prototype E-textile. The selected commercial products reflected a spectrum of price ranges, brands, and types of functionalities to capture how different companies have considered sustainable approaches in their E-textile product portfolio. The teardown analysis provided a reflection of the different materials and construction methods used in this space and provided a base for analyzing potential future improvements. The work has considered reusability and reparability from the design stage and has provided suggestions to optimize components.

The repairability of E-textiles products was identified as a crucial factor as there is no current specific legislation for recycling this product category. Hence, an effective way to increase the longevity of E-textiles would be to design them for easy reparability, component replacement, and disassembly. The workshop and a tear-down analysis were used to modify an E-textile prototype, highlighting the need for modularity, using standardized components and connectors to facilitate easy repairs and upgrades. It was believed that this approach may extend the lifespan of electronics-embedded garments or accessories and reduce electronics waste by minimizing the need for the complete disposal of the product when a small part or component fails. This approach to analyzing an E-textile prototype’s limitations in terms of sustainability and using this insight to improve them is, to the knowledge of the authors, novel.

Significant work is still required in this area to ensure that new E-textile products are constructed and disposed of in a responsible way. Immediate future work should explore expanding the range of products analyzed using this framework to identify common trends and engineering challenges in the current construction of E-textiles and to understand where improvements must be made. The sustainability of operating E-textiles must also be explored. While most devices currently use rechargeable batteries as their power source, strives have been made toward the creation of energy-harvesting capabilities within textiles [35]. The decision to incorporate such devices however must also consider the through-life energy saving of the product and the increased difficulty to recycle such devices.

The new methodology followed in this study builds on the previous tear-down workshops conducted in the field [30], and Kuusk’s framework [31], and uses an interaction matrix as a novel means of analysis. The utility of this method is proven through the analysis of three commercial products, and this will be helpful for future E-textile designers when improving commercially available and research-based E-textile products and prototypes. The approach introduced in this study can be employed in assessing any E-textile product’s impact on the environment and identifying improvement areas in an effective manner. It is recommended to use this framework in the product design stage where critical decisions are taken on the product system, materials, construction, and functionality. It will ensure proactive environmentally conscious decision-making during the E-textile’s product development. Future E-textile designs should incorporate these sustainable principles from the product design stage, to ensure that products not only meet consumer needs but also align with broader environmental and sustainability targets. With this, the industry can move towards creating more responsible, durable, and eco-friendly electronic textiles that contribute positively to both society and the environment. The information gained through this study will also be valuable when developing standards in this space. It is essential to have proper legislation in place as this market sector is growing quickly.

## Figures and Tables

**Table 1:** Interaction matrix for the heating scarf. The color code is used to indicate perceived issues with sustainability. Black: can be reused or disposed of safely. Orange: unsustainable components that can be replaced with sustainable options. Red: most critical component, deciding the sustainability of the whole product. Black background colour is used to show there are no interactions between same components. X symbol is used when there are no interactions between the relevant components.

Components of the Product	Environment	Outer Fabric Layer (Brushed Synthetic)	Woven Layer	Nonwoven Layer	Conductive Thread	Wires	Push Button/Rubber Insulated Material	Nonwoven Layer Strip	Stitches/Threads	Glue Layer	USB Power Inlet
Outer fabric layer (brushed synthetic)	Can be reused/recycled		In contact	In contact	X	X	In contact	X	In contact	X	X
Woven layer	Can be reused/recycled	In contact		In contact	X	X	In contact	X	In contact	X	X
Nonwoven layer	Can be reused/recycled	In contact	In contact		X	X	X	X	In contact	X	X
Conductive thread	Can be reused/recycled	X	X	X		X	X	In contact	In contact	Touches	X
Wires	Can be reused/recycled	X	X	X	X		In contact	X	X	X	In contact
Push button/rubber insulated material	Non-biodegradable	In contact	In contact	X	X	In contact		X	In contact	X	X
Nonwoven layer strip	Can be recycled if glue can be separated	X	X	In contact	X	X	X		In contact	Touches	X
Stitches/threads	Can be reused/recycled	In contact	In contact	In contact	In contact	Bonds all together	X	In contact		Touches	X
Glue layer	Make the whole product complicated to recycle	X	X	X	X	In contact	X	X	X		X
USB power inlet	Can be reused or recycled separately as electronics waste	X	X	X	X	In contact	X	X	X	X	

**Table 2:** Interaction matrix for the light-up cap. The color code is used to indicate perceived issues with sustainability. Black: can be reused or disposed of safely. Pink: essential components to the product but not sustainable. Orange: unsustainable components that can be replaced with sustainable options. Red: most critical component, deciding the sustainability of the whole product. Black background colour is used to show there are no interactions between same components. X symbol is used when there are no interactions between the relevant components.

Components of the Product	Environment	Outer Fabric Layer (100% Cotton)	Stiffener Material (Plastic)	Inner Fabric Cotton	Wire	Stitches/Thread	LED Mounted Strip/Transparent Light Guide Material	Heat Shrinks/Polyolefin	Stiffening Nonwoven	Velcro Strap	PCB Device	Push Button	Lithium Battery	Adhesive Material
Outer fabric layer (100% Cotton)	Can be recycled if glue can be separated		Glued together	X	X	In contact	In contact	X	In contact	In contact	X	X	X	Attach together
Stiffener material (plastic)	Non-biodegradable	Glued together		Glued together	X	In contact	In contact	X	X	X	X	X	X	Attach together
Inner fabric Cotton	Can be recycled if glue can be separated	X	Glued together		X	In contact	In contact	X	In contact	Touches	X	X	X	Attach together
Wire	Can be recycled/reused	X	X	X		In contact	Attached together	In contact	X	X	In contact	X	X	X
Stitches/thread	Can be recycled/reused	Attach together	Attach together	Attach together	In contact		In contact	X	In contact	In contact	X	X	X	X
LED mounted strip/transparent light guide material	Non-biodegradable	In contact	In contact	In contact	Attached together	In contact		In contact	X	X	X	X	X	X
Heat shrinks/Polyolefin	Non-biodegradable	X	X	X	In contact	X	In contact		X	X	X	X	X	X
Stiffening nonwoven	Can be recycled/reused	In contact	X	In contact	X	In contact	X	X		Touches	X	X	X	Touches
Velcro strap	Can be recycled/reused	In contact	X	Touches	X	In contact	X	X	Touches		X	X	X	X
PCB device	Can be reused, recycled separately as electronics waste	X	X	X	In contact	X	X	X	X	X		Holds	Holds	X
Push button	Can be reused, recycled separately as electronics waste	X	X	X	X	X	X	X	X	X	Holds		X	X
Lithium battery	Safely dispose	X	X	X	X	X	X	X	X	X	Holds	X		X
Adhesive material between layers	Make the whole product complicated to recycle	Attach together	Attach together	Attach together	X	X	X	X	Touches	X	X	X	X	

**Table 3:** Interaction matrix for the heart rate monitoring band. The color code is used to indicate perceived issues with sustainability. Black: can be reused or disposed of safely. Pink: essential components to the product but not sustainable. Orange: unsustainable components that can be replaced with sustainable options. Red: most critical component, deciding the sustainability of the whole product. Black background colour is used to show there are no interactions between same components. X symbol is used when there are no interactions between the relevant components.

Components of the Product	Environment	Outer Fabric Layer (Polyester)	Elastic Strap	Plastic Strap Adjusters	Woven Fabric Strip	Signal Transmit Conductor Strip	Insulating and Waterproof Layer	Exo Material Inner Layer	Multiple Adhesive Layers	Snap Buttons	Outer Most Layer	Electronic Module	Coin Cell Battery	PU Label
Outer fabric layer (Polyester)	Can be reused if glue can be separated		Glued together	In contact	X	X	Bonded	X	Glued together	In contact	In contact	In contact	X	In contact
Elastic strap	Difficult to recycle due to Elastomeric yarn	Glued together		In contact	Glued together	Glued together	Bonded	Glued together	In contact	Touches	Glued together	X	X	Holds
Plastic strap adjusters	Non-biodegradable	In contact	In contact		X	X	X	X	X	X	X	X	X	X
Woven fabric strip	Can be reused	X	Glued together	X		Glued together	Bonded	X	In contact	Touches	X	X	X	X
Signal transmit conductor strip	Polymer is not very degradable	X	Glued together	X	Glued together		Bonded	Attached together	In contact	In contact	X	X	X	X
Insulating and waterproof layer	Makes the whole product complicated to recycle	Bonded	Bonds all together	X	Bonds all together	Bonds all together		Bonds all together	In contact	Touches	In contact	X	X	X
Exo material inner layer	Vinyl polymer is not degradable	X	Glued together	X	X	Attached together	Bonds all together		In contact	Touches	X	Touches	X	X
Multiple adhesive layers	Makes the whole product complicated to recycle	Glued together	Bonds all together	X	Bonds all together	Bonds all together	Bonds all together	Bonds all together		Touches	Bonds all together	X	X	X
Snap buttons	Can be reused, recycled separately	In contact	Riveted to the strap	X	Touches	In contact for data transmit	Touches	Touches	Go through the layers		In contact	Holds	X	X
Outer most exo layer with laser cut design	Vinyl polymer is not degradable	In contact	Glued together	X	X	X	In contact	X	Bonds all together	In contact		X	X	X
Electronic module	Can be reused, recycled separately as electronics waste	In contact	X	X	X	X	X	Touches	X	Connected to	X		Holds	X
Coin cell battery	Safely dispose	X	X	X	X	X	X	X	X	X	X	Inserted in		X
PU label	Can be harmful to the environment when decomposing	In contact	Attaches	X	X	X	X	X	X	X	X	X	X	

**Table 4:** Interaction matrix for the prototype temperature sensing sock. The color code is used to indicate perceived issues with sustainability. Black: can be reused or disposed of safely. Orange: unsustainable components that can be replaced with sustainable options. Black background colour is used to show there are no interactions between same components. X symbol is used when there are no interactions between the relevant components.

Components of the Product	Environment	Outer Fabric Layer (Polyester Mix)	Polyester Yarn Braid	Fine Copper Wires	UV Curable Resin	Thermistors	Solder	Resistor	Threads/Stitches	Electronic Module	Battery	Connector	Silicone Cover
Outer fabric layer (Polyester and elastomeric)	Can be recycled if elastomeric compound can be separated		In contact	X	X	X	X	X	In contact	X	X	In contact	In contact
Polyester yarn braid	Can be reused/recycled	In contact	Thermistors	In contact	In contact	X	X	X	In contact	X	X	X	Touches
Fine Copper wires	Can be reused/recycled	X	In contact		In contact	In contact	In contact	In contact	X	In contact	X	X	In contact
UV curable resin	Less-biodegradable	X	In contact	In contact		In contact	In contact	In contact	X	X	X	X	X
Thermistors	Can be reused, recycled separately as electronics waste	X	X	In contact	In contact		In contact	X	X	X	X	X	X
Solder	Can be reused, once melted	X	X	Attaches	In contact	Attaches		Attaches	X	X	X	X	X
Resistor	Can be reused, recycled separately as electronics waste	X	X	In contact	In contact	X	Attached		X	X	X	X	X
Threads/Stitches	Can be reused/recycled	In contact	In contact	X	X	X	X	X		X	X	X	X
Electronic module	Can be reused, recycled separately as electronics waste	X	X	In contact	X	X	X	X	X		In contact	Holds	In contact
Battery	Can be reused, recycled separately as electronics waste	X	X	X	X	X	X	X	X	In contact		X	In contact
Connector	Can be reused, recycled separately as electronics waste	In contact	X	X	X	X	X	X	X	Holds	X		In contact
Silicone cover	Less-biodegradable	In contact	Touches	In contact	X	X	X	X	X	In contact	In contact	In contact	

## Data Availability

The original contributions presented in the study are included in the article, further inquiries can be directed to the corresponding author.
